# The natural variance of the Arabidopsis floral secondary metabolites

**DOI:** 10.1038/sdata.2018.51

**Published:** 2018-04-03

**Authors:** Takayuki Tohge, Monica Borghi, Alisdair R. Fernie

**Affiliations:** 1 Max-Planck-Institute of Molecular Plant Physiology, 14476 Potsdam-Golm, Germany; 2 Graduate School of Biological Sciences, Nara Institute of Science and Technology, Ikoma, Nara, 630-0192, Japan

**Keywords:** Secondary metabolism, Natural variation in plants, Metabolomics

## Abstract

Application of mass spectrometry-based metabolomics enables the detection of genotype-related natural variance in metabolism. Differences in secondary metabolite composition of flowers of 64 *Arabidopsis thaliana* (Arabidopsis) natural accessions, representing a considerable portion of the natural variation in this species are presented. The raw metabolomic data of the accessions and reference extracts derived from flavonoid knockout mutants have been deposited in the MetaboLights database. Additionally, summary tables of floral secondary metabolite data are presented in this article to enable efficient re-use of the dataset either in metabolomics cross-study comparisons or correlation-based integrative analysis of other metabolomic and phenotypic features such as transcripts, proteins and growth and flowering related phenotypes.

## Background and Summary

Plant secondary metabolites (so-called specialized metabolites) that have high natural diversity in their chemical structures and abundances can be identified through metabolic screening of populations even in the comparisons between ecotypes and cultivars belonging to the same species^1–3^. This may represent relatively recent adaptations or more phylogenetical restrictions in the evolution of such metabolisms^3–5^. With metabolomic screening of such populations, metabolic polymorphism in aliphatic glucosinolates^6^, flavonol-glycosides^7^ and phenylacylated-flavonols^3^ have been discovered in Arabidopsis. Additionally, a key gene of production of phenylacylated-flavonols for the conferral of protection towards UV irradiation^3^, was characterized by an integrative functional genomic approach. Since several physiological studies using Arabidopsis accessions have been reported with phenotypic analysis under stress conditions such as UV-B irradiation^8^, drought and salinity stress^9,10^ and biotic stressors^11^, understanding of plant secondary metabolites for the conferral of protection towards stress condition is highly important. To capture the variance of secondary metabolites across populations, liquid chromatography-mass spectrometry (LC-MS) has often been preferred to other analytical methods as it presents the technical advantage of capturing the most extensive variety of plant metabolites.

Here, data of floral secondary metabolite abundance measured in a population of 64 *Arabidopsis thaliana* (Arabidopsis) natural accessions are presented (Data Citation 1)(Data Citation 2). Sixty-eight secondary metabolites were measured via LC-MS, ions acquired in positive and negative ion detection mode, and compounds annotated through a combination of chemical confirmation with analytical standards and comparative analysis with flavonoids knockout and over-expresser Arabidopsis lines^12,13^. The list of the Arabidopsis accessions used in this study, and raw and normalized metabolomics data are provided (Data Citation 1)(Data Citation 2), respectively. This dataset can be used for cross-study comparisons of plant metabolites, investigations on the reproducibility of metabolomics data, and in-depth analysis of plant metabolism. Importantly, transcriptomics data obtained from 10 samples in this experimental set is available in the Gene Expression Omnibus (GEO) database (Data Citation 3). Correlation studies with data of metabolomics, transcriptomics, proteomics and phenomic data of floral related traits are also anticipated. In addition, the presence in this dataset of standard reference files and complex biological data files, which were acquired on the same LC-MS system, makes it useful for practical exercises on data analysis and interpretation. Finally, as several secondary compounds initially identified in model plants bring nutritional and health benefits to humans^14,15^, these data will be helpful in the design of future plant metabolic engineering used for translational genomics applications from model species to crops.

## Methods

### Plant material and sample preparation

Seeds of Arabidopsis natural accessions (Table 1 (available online only)) were germinated on 1/2 MS salts solidified with 1% of agar in a growth chamber (16 h light, 140-160 μmol m^−2^ s^−1^, 20 °C; 8 h dark, 16 °C) after vernalization (two days in the dark at 8–10 °C). Fourteen days after planting, the seedlings were transferred onto soil (GS-90 Einheitserde; Gebrueder Patzer) and grown in a greenhouse (16 h light, an average irradiance of 120 μmol m^−2^ s^−1^, 20 °C; 8 h dark, 16 °C) until flowering. Positioning of the plants was randomized during plant growth. Fully open mature flowers (first flowers) were harvested at around noon (after approximately 5 h of light) and immediately frozen in liquid nitrogen for further analysis. Flowers from three plants were individually harvested to prepare one biological replicate. Sample preparation and extraction were performed as previously described^3^.

### LC-MS analysis and flavonoid mutant-based peak annotation

Profiling of secondary metabolites was performed as previously described^3,16^. Briefly, flower tissues were ground with liquid nitrogen and homogenized in a mixer mill for 3 min at 25 *Hz* with a zirconia bead and 20 μL of extraction buffer (80% methanol, prepared with 5 μg mL^−1^ isovitexin as an internal standard) per mg of ground tissue (e.g., 204.0 μl extraction buffer for 10.2 mg fresh weight sample). Thereafter, the supernatant was separated from the cellular debris via centrifugation at 12,000 x G and 3 μL of the clarified supernatant directly injected in an HPLC system Surveyor (Thermo Finnigan, USA) coupled to LTQ-XP system (Thermo Finnigan, USA) for metabolite profiling described as below. All samples including flower extracts obtained from Arabidopsis mutants described in ‘Data processing and metabolite data analysis’ were analyzed together. Sample run order was determined by replicates consecutively.

### Chromatography

Chromatography was performed as previously described^16^. Samples were run on a Surveyor HPLC system (Thermo, USA), 150×2 mm, 2.0 μm particles (Reverse Phase Luna C18_(2)_, Phenomenex, USA), HPLC column at 28 °C oven temperature. The solvents used for the assay consisted of water containing 0.1% *v*/*v* formic acid (Solvent A) and an acetonitrile solution containing 0.1% *v*/*v* formic acid (Solvent B). Gradient [time (min)/%B] starting: 2.0/0, 4.0/15, 14.0/32, 19.0/50, 19.01/100, 21.0/100, 21.01/0, 23.0/0 at flow rate 0.20 mL^ ^min^−1^. Injection volume was 2 μL.

### Mass spectrometry

The compounds were detected using a Thermo LTQ-XL Linear-Ion-Trap mass spectrometer (expected resolution is 0.3 u FWHM) with electrospray ionization (ESI) mode in negative (collision energy: 0 and 30 meV) and positive ion detections with a scan range from 100–2000 *m/z*. Main MS parameters (capillary temperature: 275 °C; source voltage: 4.00 kV(negative) and 4.50 kV (positive); capillary voltage: −50 V(negative) and 50 V(positive) were optimized for the detection of plant secondary metabolism. Other MS parameters are described in Tohge *et al.*, 2010^16^. The LTQ-XP used the Xcalibur software (Thermo Finnigan, USA) version 2.1.0 for data acquisition.

### Data processing and metabolite data analysis

Data were processed using Xcalibur 2.1.0 software, and peak identification and annotation implemented through a combination of the following approaches: standard chemical confirmation^17^, MS fragmentation and retention time profiling, mutant analysis^3,12,13^, literature/database survey^18,19^. The following Arabidopsis mutants known for having altered flavonoid profiling were used as control lines for the determination of flavonoid derivatives: *UDP-glucosyl transferase 78D2* (*ugt78d2*), decreased production of flavonoid-3-*O*-glucoside^20^; *transparent testa* 7 *(tt7)*, no production of quercetin and isorhamnetin derivatives^21^; *ugt78d1,* no production of flavonol-3-*O*-rhamnosides^22^; *ugt78d3*, no production of flavonol-3-*O*-arabinosides^23^; *O-methyltransferase 1 (omt1),* no production of isorhamnetin-derivatives^12^; *ugt89c1*, no production of flavonol-7-*O*-rhamnosides^24^; *tt4*, no production of all flavonoids^25,26^; *production of anthocyanin pigment 1-Dominant (pap1-D)*, increased accumulation of anthocyanins^20,27^. Peak picking was performed by Xcalibur Quan Browser (Window (sec), 30; highest peak; minimum peak height (S/N), 3.0; Baseline window, 80-150; area noise factor, 2; peak noise factor, 10; peak height (%), 5.0, tailing factor, 1.5).

### Transcriptomic data

Transcriptomic analysis was performed using ATH1 microarrays as described previously^3^ with ten accessions (Col-0, C24, Cvi, Da, Rsch, Ler-0, Ws, Sap, Stw and RLD). Duplicate hybridizations were carried out for Col-0 and C24, and a single hybridization was performed for all the other accessions except Col-0 and C24. Data is deposited in the Gene Expression Omnibus database (Data Citation 3).

## Data Records

Raw data obtained from the analysis of natural Arabidopsis accessions and mutant reference lines have been deposited in the Metabolights (Data Citation 1). Raw data contains two negative (collision energy: 0 and 30 meV) and one positive ion detections. Cdf files contain negative and positive ion detections without data of in-source fragmentation using collision energy. This dataset contains a total of 216 raw files resulting from 72 lines (64 accessions and 8 Arabidopsis mutant lines) with three biological replicates each. A dataset of floral secondary metabolite (68 compounds; 16 glucosinolates, 3 hydroxycinnamate derivatives, 42 flavonol derivatives and 7 putative polyamines) and general statistics relative to the natural accessions used in the study is provided (Data Citation 2). Metabolite data was obtained from a dataset previously published^3^ and reformatted for correlation-based analysis by average-scaling and log-transformation ([log_2_(mean(replicates)/mean(mean of all accessions)]) (Data Citation 2). The geographic coordinates of the Arabidopsis accessions provided in Table 1 (available online only) are updated accordingly with the Arabidopsis 1001 genome database (http://1001genomes.org/)^28^.

## Technical Validation

To qualitatively and quantitatively validate metabolite data obtained from three biological samples the standard deviation was estimated (Data Citation 2).

## Usage notes

**Figure 1:**
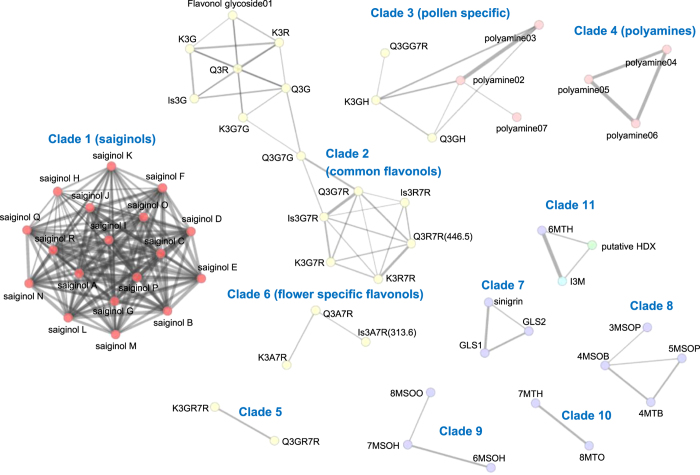
Correlation network of Arabidopsis floral secondary metabolites. Network analysis and visualization were performed with Cytoscape using an organic layout. The Pearson correlation threshold of 0.6 was chosen to determine the connections between edges and nodes. Nodes represent metabolites and the edges the interaction between metabolites. The size of nodes and edges maps to clustering coefficient and correlation coefficient, respectively, with small nodes and thin edges representing small values. Different classes of metabolites are represented with different colors: saiginols, red; flavonols, yellow; polyamine, pink; purple, aliphatic glucosinolates; green, putative hydroxycinnamate; light blue, indole glucosinolate.

Data of floral secondary metabolites are presented in Excel files (Data Citation 2). For each compound, the method used for peak identification/annotation, which includes retention time, ion detection mode and relative peak area, is specified. The value of the relative peak area was obtained from the average of three measurements (*n*=3) normalized by the standard deviation (SD)(Data Citation 2). Compound’s family name and reference literature are also provided. The abundance of floral metabolites, normalized by average-scaling (mean/average) and log-transformation (log_2_) is reported (Data Citation 2). The dataset here presented can be used for cross correlation studies to integrate metabolomics with transcriptomics, proteomics, and floral phenotypic data. Figure 1 shows an example of metabolite-metabolite correlation network analysis (*r*^2^>0.6, Pearson correlation estimated R statistical package (https://www.r-project.org/)) performed with the data reported (Data Citation 2). Visualization of network connection based on coefficient value was performed with Cytoscape (http://www.cytoscape.org/) using an organic layout style (Data Citation 2). As previously discussed^3^ accession-specific floral phenylacyl-flavonol glycosides (saiginols, indicated with the number 1 in Fig. 1) show a strong correlation within the saiginol clade. The following ten additional clades of compounds were also identified and these are indicated in Fig. 1 with the following numbering: 2) common flavonol mono- or di-glycosides, 3) pollen specific flavonols and pollen specific polyamines, 4) putative pollen specific polyphenolic polyamines, 5) flavonol-3-*O*-(2′′-*O*-rhamnosyl)glucoside-7-*O*-rhamnosides, 6) flower specific flavonol-glycosides, 7) accession-specific glucosinolate, 8) short-chain aliphatic glucosinolates, 9), long-chain aliphatic sulfinyl-glucosinolates, 10) long-chain aliphatic thio-glucosinolates, and 11) other glucosinolates as for example indolic glucosinolates. No subclades of hydroxycinnamates were found. Network analysis suggests that metabolites that belong to the same clade are produced in Arabidopsis natural accessions that share the common genetic polymorphism, transcriptionally co-regulated, or are the resulted of a similar metabolic pattern maintained by the combination of different metabolic flux changes. The data presented in this article are useful in biodiversity studies, e.g., to investigate relationships between natural metabolic diversity and accession distribution, physiological diversity and the genomic polymorphism.

## Additional information

**How to cite this article**: Tohge, T, et al. The natural variance of the Arabidopsis floral secondary metabolites. *Sci. Data* 5:180051 doi: 10.1038/sdata.2018.51 (2018).

**Publisher**’**s note**: Springer Nature remains neutral with regard to jurisdictional claims in published maps and institutional affiliations.

## Supplementary Material



## Figures and Tables

**Table 1:** The site of origin of Arabidopsis accessions presented in this article.

**No.**	**Accession No.**	**Name**	**city**	**country**	**altitude**	**latitude**	**longitude**
1	CS28017	An-2	Antwerpen	Netherlands	9.0	51.58	4.35
2	CS28018	Ang-0	Angleur	Belgium	270.4	50.61	5.59
3	CS76445	Bd-0	Berlin/Dahlem	Germany	47.3	52.46	13.29
4	CS28062	Be-0	Bensheim/Bergstr.	Germany	99.4	49.68	8.61
5	CS28086	Bla-11	Blanes/Gerona	Spain	95.5	41.69	2.80
6	CS76098	Blh-1	Bulhary	Austria	248.4	48.83	16.74
7	CS28100	Bsch-2	Buchschlag/FFM	Germany	95.9	50.02	8.67
8	CS28102	Bu-2	Burghaun/Rhon	Germany	465.4	50.69	9.72
9	CS76105	Bur-0	Burren (Eire)	ireland	186.2	53.20	-8.98
10	CS76106	C24	Lousa	Portugal	146.9	40.11	-8.244
11	CS76109	Can-0	de Gran Canaria	Morocco	1260.0	29.21	-13.48
12	CS28133	Cha-0	Champex	Switzerland	1973.8	46.02	7.07
13	CS28164	Co-3	Coimbra	Portugal	146.9	40.20	-8.42
14	CS76113	Col-0	Columbia	USA	249.4	38.30	-92.30
15	CS76116	Cvi-0	Cape Verdi Islands	Senegal	1150.0	15.11	-23.62
16	CS28200	Da-0	Darmstadt	Germany	89.1	49.87	8.65
17	CS28206	Dijon-M		Russia	186.3	55.45	37.35
18	CS76126	Edi-0	Edinburgh	UK	64.8	55.97	-3.22
19	CS76479	El-0	Ellershausen	Germany	492.7	51.51	9.682
20	CS76127	Est-1	Estland	Estonia	29.1	58.30	25.30
21	CS28973	Gol-1	Scotland Golspie	UK	6.7	57.97	-3.97
22	CS76137	Gr-1	Graz, Austria	Austria	332.0	47.00	15.50
23	CS28349	HI-3	Holtensen	Germany	260.3	52.14	9.38
24	CS28351	HOG	Khodga-Obi-Garm	Tajikistan	1750.0	38.55	68.47
25	CS76145	Hs-0	Hannover/Stroehen	Germany	39.1	52.50	9.50
26	CS28365	Je-54		Czech republic	279.0	49.30	17.00
27	CS76148	JEA	St Jean Cap Ferrat	France	10.8	43.68	7.33
28	CS79018	Kas-1	Kashimir	India	2324.0	35.00	77.00
29	CS28389	Kl-0	Koeln	Germany	414.1	50.95	6.97
30	CS28395	Kn-0	Kaunas	Lithuania	87.0	54.90	23.89
31	CS77020	L*er*-0	Landsberg	Germany	66.5	47.98	10.87
32	CS76168	Lip-0	Lipowiec/Chrzanow	Poland	240.1	50.00	19.30
33	CS76175	Lov-5	Lovvik	Sweden	2.60	62.80	18.08
34	CS28922	Lovel-1	Løvel	Denmark	3.0	56.57	9.48
35	CS77056	Lu	Lund	Sweden	13.1	55.70	13.20
36	CS28493	Mh-1	Muehen (OstPr)	Poland	193.2	53.31	20.12
37	CS76192	Mt-0	Martuba/Cyrenaika	Libya	283.1	32.34	22.46
38	CS28528	Nd	Niederzenz	Germany	49.1	47.40	8.18
39	CS76199	NFA-8	Ascot (England)	UK	79.9	51.41	-0.64
40	CS28564	No-0	Nossen	Germany	417.7	51.06	13.30
41	CS1402	Nok-2	Noordwijk	Netherlands	7.9	52.25	4.45
42	CS28568	Nok-1	Noordwijk	Netherlands	7.9	52.25	4.45
43	CS28576	Nw-3	Neuweilnau	Germany	457.8	50.31	8.40
44	CS28583	Old-1	Oldenburg	Germany	9.3	53.17	8.20
45	CS76203	Oy-0	Oystese	Norway	31.0	60.39	6.19
46	CS76211	Petergof	Petergof	Russia	153.3	59.87	29.91
47	CS28648	Po-0	Poppelsdorf	Germany	72.1	50.72	7.09
48		Pyl-1	Le Pyla	France	45.4	44.65	-1.17
49	CS76216	Ra-0	Randan	France	305.8	46.00	3.30
50	CS76588	RLD-1		Netherlands	17.8	52.15	5.30
51	CS28715	Rsch-0	Rschew/Starize	Russia	231.7	56.20	34.30
52	CS28718	Rubezhno-1	Rubezhnoe	Ukraine	189.2	49.00	38.30
53	CS76224	Sap-0	Slapy	Czech republic	494.6	49.49	14.24
54	CS28725	Sav-0	Slavice	Czech republic	701.0	49.18	15.88
55	CS28735	Shakdara	Shakdara River	Tajikistan	4178.1	39.25	68.24
56	CS76231	St-0	Stockholm	Sweden	76.7	59.00	18.00
57	CS76605	Stw-0	Stobowa/Orel	Russia	217.0	52.00	36.00
58	CS76242	Ta-0	Tabor	Czech republic	398.2	49.50	14.50
59	CS28757	Te-0	Tenala	Finland	30.9	60.06	23.30
60	CS28786	Ty-0	Taynuilt	UK	19.3	56.43	-5.23
61	CS28817	Wei-1	Weiningen	Switzerland	529.4	47.41	8.42
62	CS28819	Will	Vilnius	Lithuania	147.9	54.68	25.32
63	CS76303	Ws-0	Wassilewskija	Russia	129.0	52.30	30.00
64	CS28847	Zue-1	Zurich	Switzerland	707.6	47.37	8.55
Geographical coordinates were obtained from a previous study^3^ and the 1001 genomes project (http://1001genomes.org/).							
